# Transcriptomic Screening of *Alternaria oxytropis* Isolated from Locoweed Plants for Genes Involved in Mycotoxin Swainsonine Production

**DOI:** 10.3390/jof10010088

**Published:** 2024-01-22

**Authors:** Shuangjie Yuan, Qingmei Zhao, Kun Yu, Ying Gao, Zhengbing Ma, Huanyu Li, Yongtao Yu

**Affiliations:** 1 School of Animal Science and Technology, Ningxia University, Yinchuan 750021, China; 2 Ningxia Key Laboratory of Ruminant Molecular and Cellular Breeding, School of Animal Science and Technology, Ningxia University, Yinchuan 750021, China; 3 College of Biological Science and Engineering, North Minzu University, Yinchuan 750021, China

**Keywords:** locoweed, *Alternaria oxytropis*, transcriptome, swainsonine, differentially expressed genes

## Abstract

Locoweed is a collective name for a variety of plants, such as *Oxytropis* and *Astragalus* L. When these plants are infected by some fungi or endophytes, they will produce an alkaloid (swainsonine) that is harmful to livestock. Chronic toxicity characterized by neurological disorders occurs in livestock overfed on locoweed, and swainsonine (SW) is considered a major toxic component. The mechanism of the SW synthesis of endophytic fungi from locoweed remains unknown. In order to further discover the possible synthetic pathway of SW, in this study, a mycotoxin (SW) producer, *Alternaria oxytropis* isolate, UA003, isolated from Locoweed plants, and its mutant were subjected to transcriptomic analyses to ascertain the genes involved in the synthesis of this toxin. Mutant strain *A. oxytropis* E02 was obtained by ethyl methanesulfonate (EMS) mutagenesis treatment, and the strains were sequenced with different culture times for transcriptomic analysis and screening of differentially expressed genes. The results show a highly significant (*p* < 0.01) increase in SW yield in the *A. oxytropis* E02 strain obtained by EMS mutagenesis treatment compared to *A. oxytropis* UA003. A total of 637 differentially expressed genes were screened by transcriptome sequencing analysis, including 11 genes potentially associated with SW biosynthesis. These genes were screened using GO and KEGG data annotation and analysis. Among the differential genes, evm.TU.Contig4.409, evm.TU.Contig19.10, and evm.TU.Contig50.48 were associated with L-lysine biosynthesis, the L-pipecolic acid pathway, and the α-aminoadipic acid synthesis pathway. This study provides new insights to elucidate the mechanism of SW synthesis of endophytic fungi in locoweed and provides data support for further exploration of *A. oxytropis* genomics studies.

## 1. Introduction

Locoweeds are some poisonous species of the genus *Oxytropis* and *Astragalus* L. They are widely distributed in the grasslands of arid and semi-arid regions in China, the United States, and Canada. Chronic toxic diseases characterized by clinical symptoms such as ataxia, muscle paralysis, abortion, and infertility can occur in livestock with excessive consumption of locoweeds, which brings important economic losses to affected areas [1]. When these plants are infected by some fungi or endophytes, they will produce an alkaloid that is harmful to livestock. The indolizidine alkaloid swainsonine (SW) is the main toxic component of locoweeds, which specifically inhibits lysosomal α-mannosidase and Golgi mannosidase II in mammalian cells and disrupts the intracellular membrane system [2,3]. There is sufficient evidence to show that endophytic fungi of locoweeds, *Alternaria* section *Undifilum* spp., including *Alternaria oxytropis*, *A. fulvum,* and *A. cinereum*, are responsible for the biosynthesis of SW [4,5,6,7] and are closely related to the content of SW in locoweeds [8,9,10,11].

The putative biosynthesis pathways of SW have been described in the plant pathogenic fungus *Slafractonia legumincola* and the entomopathogenic fungus *Metarhizium anisopliae* [12,13,14]. Lysine, saccharopine, and L-pipecolic acid are key precursers or intermediates in the biosynthetic pathway of SW in these fungi. Increasing evidence suggests that *Alternaria* section *Undifilum* spp. may share some SW biosynthetic steps similar to those of *S. legumincola* and *M. anisopliae* [12,15,16,17], but the specific functions of these genes and their roles in SW synthesis in the fungus *Alternaria* sect. *Undifilum* spp. are still unclear. Recent studies have shown that fungi capable of producing SW, including the plant pathogenic fungus *S. leguminicola*, entomopathogenic fungus *Metarhizium robertsii*, plant epiphytic fungus Chaetothyriaceae spp. of the genus *Ipomoea*, the human and animal dermatopathogenic fungi *Trichophyton* spp. and *Arthroderma* spp., etc., all have a highly homologous and structurally similar cluster of type I polyketides synthetase (T1-PKS) secondary metabolite synthesis genes, and these genes in the cluster may encode enzymes or proteins required for the chemical reactions involved in the L-pipecolic acid-to-SW synthesis pathway, and are thus named the swainsonine biosynthesis gene cluster (SWN) [8,16]. However, the function of this gene cluster in the endophytic fungus *A. oxytropis* in locoweed remains unproven. A recent study suggests that transcriptional regulatory molecules such as C2H2 may be involved in the regulation of swnk gene expression, a key gene in the SWN gene cluster [18], but the exact mechanism of action needs to be further investigated.

At present, the mechanism of SW synthesis is still not elucidated, though the elucidation of this mechanism can lay the foundation for artificially controlling the synthesis of SW by endophytic fungi and the detoxification and utilization of locoweed. The regulation of transcription levels plays an important role in the regulation of gene expression related to the synthesis of fungal secondary metabolites [19]. In recent years, the transcriptional regulation mechanism of secondary metabolite synthesis has become a research hotspot in the field of fungi. For example, the transcription factor *hmgR* of *Penicillium marneffei* affects the production of melanin precursors by specifically regulating the expression of tyrosine synthesis-related genes [20]. *Trichoderma arundinaceum* transcription factors *tri6* and *tri10* can regulate the expression of related genes in the monosporene synthesis gene cluster, thus affecting the synthesis of monosporene HA and its precursors [21].

The above research provides a new idea for the study of SW synthesis mechanisms in locoweed endophytic fungi, and the study of transcriptional regulation mechanisms related to SW synthesis in locoweed endophytic fungi can lay the foundation for further clarifying the interaction between enzyme coding genes involved in chemical reactions and the expression regulation genes of these genes and their transcriptional regulation networks.

In this study, the chemical mutagen ethyl methanesulfonate (EMS) was used to treat the wild-type strain *A. oxytropis* UA003, isolated in vitro from the endophytic fungus of locoweed, and then further screened for mutant strains with significant changes in the synthesis of swainsonine. EMS is a chemical mutagen that can induce point mutations in DNA by modifying the bases, especially guanine. Then, the transcriptome sequencing analysis of the wild-type strain and the mutant strain was carried out using high-throughput sequencing technology to screen the genes that might be involved in SW synthesis. This study provides an important theoretical basis for further elucidation of the mechanism of SW synthesis by the endophytic fungus *A. oxytropis* in locoweed.

## 2. Materials and Methods

### 2.1. Experimental Strains, Instruments, and Reagents

*A. oxytropis* strain UA003 was isolated from *Astragalus variabilis* [18]. The strain was incubated on potato dextrose agar (PDA) plates at 25 °C for 30 days. SimpliNano ultra-micro spectrophotometer (GE HealthCare, GE Healthcare Technologies Inc., Chicago, IL, USA), qTOWER fluorescence quantitative PCR instrument (Jena, Analytik Jena (Beijing) Instruments Co., Ltd., Beijing, China), total RNA extraction kit (TaKaRa, TaKaRa Biotechnology (Dalian) Co., Ltd., Dalian, China), primeScript RT reagent kit with gDNA eraser, and B GreenTM Premix Ex TaqTM II enzyme (TaKaRa, TaKaRa Biotechnology (Dalian) Co., Ltd., China) were used in this study. Snail enzyme, lysing enzyme, cellulase, EMS (Sigma Aldrich Trading Co., Ltd. Shanghai, China), anhydrous ethanol, trichloromethane, isopropanol (Sinopharm, Sinopharm Group Co., Ltd., Shanghai, China), and DEPC (TianGen, TIANGEN BIOTECH (BEIJING) Co., Ltd., Beijing, China) were also used in this study. The primers used were synthesized by Sangon Biotech (Sangon Biotech (Shanghai) Co., Ltd., Shanghai, China).

### 2.2. Preparation of Protoplasts

The wild-type strain *A. oxytropis* UA003 was inoculated onto PDA medium and incubated at 25 °C for 30 days. The resultant fungal colonies were punched into 6 mm-diameter cakes, which were subsequently inoculated into conical flasks containing 100 mL of PDB medium and incubated at 25 °C for 15 days with a thermostatic oscillator. The fungal culture was passed through a Miracloth, then the mycelia were washed with a 0.6 mol/L MgSO_4_ solution and subsequently dried with sterilized absorbent paper. After grinding 100 mg of mycelium with a mortar, it was transferred to a 50-milliliter sterile centrifuge tube. Ten milliliters of enzymatic solution, consisting of cellulase, lysing enzyme, and snail enzyme, with a mass fraction ratio of 1:1:1:1.2, were added to a centrifuge tube at a mass–volume ratio of 1:10. The mixture was then incubated at room temperature with a rotation rate of 90 revolutions per minute for 2 h. The mycelium underwent enzymatic digestion before being filtered using a Miracloth. The resulting filtrate was then transferred to a new 50-milliliter centrifuge tube and centrifuged at ambient temperature for 10 min at 4000 rpm. This mixture was subsequently centrifuged for 10 min at 4000 rpm. The supernatant was extracted using a small pipette, and the cells were resuspended in the centrifuge tube by adding 10 mL of STC buffer. After discarding the supernatant, 1 mL of STC buffer was added to the centrifuge tube to resuspend the cells. Then, 20 microliters of the suspension were transferred to a hemocytometer for cell counting under a microscope. Next, the concentration of protoplasts was adjusted to 1 × 10^5^–1 × 10^6^ cells/mL by adding an appropriate amount of STC buffer to the centrifuge tube.

### 2.3. Protoplast Chemotaxis Treatment

We took 100 µL of protoplast suspension and added various volumes of 10 M EMS stock solution and mixed well to achieve working concentrations of 0.02, 0.04, 0.06, and 0.08 M EMS. Then, it was incubated at a constant temperature in a shaker at 90 rpm for 20 and 30 min, respectively. After that, it was centrifuged at 4000 rpm for 6 min, and the supernatant was discarded. Then, we added 100 µL of STC buffer to the centrifuge tube to resuspend the cells, and the suspension was spread evenly on the regeneration medium and incubated at 20 °C. The process was performed in three parallel treatment groups for each concentration, along with a blank control group in which EMS was not added.

### 2.4. Determination of SW and Screening of Mutant Strains

The wild strains and EMS-induced strains were inoculated on PDA medium, respectively, and cultured for 30 days. Fresh mycelium of each strain cultured on PDA medium was collected at a rate of 0.5~1 g, frozen in liquid nitrogen, ground into powder, and dried. The dried powder was placed in a 10 mL centrifuge tube containing 5 mL of methanol and extracted by ultrasonic extraction at 30 °C for 30 min. Each sample was extracted three times. The extracts were combined, and the solvent was then evaporated under reduced pressure. The crude extract was dissolved in an appropriate amount of methanol, filtered through a 0.22 μm pore size filter membrane, and the volume was adjusted to 5 mL and examined. Three parallel replicates were set up simultaneously for each strain. The SW content in the mycelium was determined using the α-mannosidase inhibition method, as reported by Ashley et al. [22]. Then, we compared the SW content in the mycelium of mutant strains and wild-type strains treated with EMS mutagenesis and screened for mutant strains with significant changes in SW content. The wild-type strain *A. oxytropis* UA003 and the screened mutant strains, which exhibited significant changes in SW content, were inoculated on PDA medium and cultured for three consecutive generations. The SW content in the mycelium of the fungi from each generation was determined to identify whether EMS mutant strains with significant differences in SW synthesis compared to the wild-type strains, which remained stable. The SW content in the mycelium of the wild-type strain and the selected mutant strains was compared after 20 and 30 days of culturing.

### 2.5. Fungal RNA Extraction and RNA Sequencing

The mycelium from 50 to 100 mg of wild-type strain *A. oxytropis* UA003 and EMS mutagenized strains exhibiting marked changes in SW content cultured for 20 and 30 days at 25 °C on PDA medium, respectively, were harvested. They were then ground into a powder using liquid nitrogen. The powder was transferred to a 2 mL centrifuge tube, and the total RNA for each group of fungi was extracted by kit following the manufacturer’s instructions. Agarose gel electrophoresis was conducted to detect any RNA degradation or contamination, while Nanodrop was utilized for RNA purity assessment. Moreover, Qubit was employed to quantify the concentration of RNA, and the integrity of RNA was evaluated using Agilent 2100. Fungal RNA samples from cultures of the wild-type strain *A. oxytropis* UA003 harvested at 20 and 30 days were separated into two groups, i.e., C20 and C30. Each group had three parallel replicates, designated as CA01, CA02, and CA03, and CB01, CB02, and CB03, respectively. Fungal RNA samples from strains mutagenized with EMS and cultured for 20 and 30 days were divided into two groups, i.e., T20 and T30. Each group consisted of three parallel replicates, labeled TA01, TA02, and TA03, and TB01, TB02, and TB03, respectively.

After qualifying the RNA samples, eukaryotic mRNA was enriched using magnetic beads with Oligo (dT) that is bound to the polyA tail of the mRNA by A-T complementary pairing. Subsequently, the mRNA was interrupted into short fragments by adding a fragmentation buffer. The cDNA first strand was synthesized in the M-MuLV reverse transcriptase system using fragmented mRNA as the template and random oligonucleotides as the primer. The initial cDNA strand was synthesized using fragmented mRNA as a template and random oligonucleotides as primers in the M-MuLV reverse transcriptase system. The RNA strand was then degraded by RnaseH, and the second cDNA strand was synthesized with dNTPs in the DNA polymerase I system. Technical abbreviations are explained upon first use. The double-stranded cDNA underwent purification, end-repair, and A-tailing procedures. Subsequently, it was ligated to the sequencing adapter and screened with AMPure XP beads to isolate fragments of approximately 200 bp. The final library was generated by PCR amplification and purification with AMPure XP beads. After constructing the library, we initially quantified it using the Qubit 2.0 Fluorometer and diluted it to 1.5 ng/μL. Next, we determined the insert size of the library with the Agilent 2100 Bioanalyzer(Agilent Technologies Inc., Santa Clara, CA, USA), and the size matched our expectations. We then used qRT-PCR to precisely quantify the effective concentration of the library (which was above 2 nM) to verify its quality. After meeting the expected insert size, we used qRT-PCR to precisely measure the effective concentration of the library (which was greater than 2 nM) and ensured its quality. After passing the library check, libraries were grouped by effective concentration, and the target downstream data volume was used for Illumina sequencing. This generated 150 bp of paired-end reads using the Sequencing by Synthesis method. Four different fluorescently labeled dNTPs, DNA polymerase, and junction primers were then added to the sequencing flow cell for amplification. When extending the complementary strand of each sequencing cluster, the addition of each fluorescently labeled dNTP released the corresponding fluorescence that was captured by the sequencer. This was then converted by computer software into sequencing peaks, and the fragment information of the sequence was obtained.

### 2.6. Bioinformatics Analysis

The obtained raw sequencing data were filtered to remove splices, reads containing greater than 10% N percentage, and low-quality reads, resulting in clean data. Q20, Q30, and GC content calculations were conducted on filtered data to evaluate the quality of the sequencing output. Technical term abbreviations are explained upon first use. The genomic alignment of the clean data sequences against the *A. oxytropis* UA003 genome was analyzed using HISAT2 (v2.0.5) software, and the read distribution across the genome was quantified. All reads were assembled with Cufflinks and compared to the reference genome using Cuffcompare for gene prediction. Variable shear events were analyzed through rMATS (3.2.5) software, and differential analysis was conducted on each category based on the number of expressed variable shear events. The tools used for chromosome coordinate sorting and reads de-duplication were compared, including samtools and Picard tools. Subsequently, mutation detection software samtools was employed for SNP calling and InDel calling, followed by filtering the results. Technical abbreviations are explained upon first use. The HTSeq software (HTSeq-count 0.9.1) was utilized to analyze the gene expression levels of each sample. The gene expression levels were compared under different experimental conditions through FPKM distribution plots of all genes and violin plots. The correlation of gene expression levels between samples was assessed using the square of Pearson’s correlation coefficient. Differential gene expression analysis was conducted for the two comparison groups using the DESeq2 R software (version 1.16.1), with a threshold of |log2(FoldChange)| > 1 and a *p*-value of <0.05 for gene screening.

The violin plot diagram was utilized to analyze the general distribution of differential genes. The FPKM values of the differential genes under varying experimental conditions were employed as expression levels for hierarchical clustering. The log2(ratios) clustering analysis using H-cluster, K-means, and SOM methods was used to analyze the relative expression levels of the differential genes. The results show that genes in the same cluster display similar expression level trends under varying treatment conditions. Additionally, a Venn diagram of the differential genes was used to illustrate the number of differentially expressed genes between comparison groups and the extent of their overlap. The goseq (version 2.12) software was utilized to examine the GO enrichment of differentially expressed genes. This process allowed for the visualization of the distribution of the number of differentially expressed genes across enriched GO terms, including those related to biological processes, cellular components, and molecular functions. The KOBAS (v2.0) software was used to analyze the KEGG enrichment of differentially expressed genes. The results are presented in a scatter plot, and the pathway map includes annotations of the differential genes. The analysis of differential gene–protein interaction networks utilized interactions from the STRING protein interaction database (http://string-db.org/, accessed on 7 November 2022). The resulting data files for the differential gene–protein interaction networks were then imported into Cytoscape software (Cytoscape 3.5.1) for visualization and editing. Technical abbreviations are explained upon their first use.

### 2.7. Analysis of Real-Time qPCR Data

Based on the transcriptome analysis of the screened fungi, primers were designed using the BLAST-Primer software (Primer Premier 5.0) available on NCBI (Supplementary Table S1). Technical term abbreviations are explained upon first use. Real-time PCR amplification reactions were performed using the B639273 2X SG Fast qPCR Master Mix (High Rox) kit from BBI (BBI Co., Ltd., Shenzhen, China). The cDNAs derived from the reverse transcription of groups C20, C30, T20, and T30 served as templates. The PCR reaction solution was prepared with GAPDH as the internal reference gene and dd H_2_O as the negative control, according to the indicated components. Three replicates were established for each sample using the SybrGreen qPCR Master Mix (2X) 10 μL reaction system, along with 0.4 μL of PCR forward primer, 0.4 μL of PCR reverse primer, 2 μL of cDNA template, and 7.2 μL of RNase Free dd H_2_O. The reaction conditions consisted of pre-denaturation at 95 °C for 3 min, denaturation at 95 °C for 15 s, annealing at 60 °C for 30 s, and 45 cycles. The samples were placed in 96-well plates and analyzed on a QuantStudioTM1 Plus fluorescence quantitative PCR instrument (Thermo Fisher, Thermo Fisher Scientific, Waltham, MA, USA). The Ct values for each group were normalized using GAPDH as an internal reference gene. The expression levels of different genes in each strain were determined using the 2^∆∆Ct^ method, where ∆∆Ct = [Ct (target gene in treatment group) − Ct (internal reference gene in treatment group)] − [Ct (target gene in control group) − Ct (internal reference gene in control group)].

### 2.8. Data Processing and Statistical Analysis

Statistical analysis was conducted using IBM SPSS Statistics 23.0 and GraphPad Prism version 8.3.0. All data from a minimum of three biological replicates are presented as the mean ± standard deviation. Unpaired *t*-tests were performed between two groups, or one-way ANOVA was used between multiple groups, to calculate *p*-values (ns, not significant; * *p* < 0.05, ** *p* < 0.01, *** *p* < 0.001, and **** *p* < 0.0001), unless otherwise indicated.

### 2.9. Data Availability

The transcriptome data on the fungi of this study have been uploaded to the NCBI GenBank Sequence Read Arxchive (SRA) database under accession number PRJNA1017857.

## 3. Results

### 3.1. Changes in SW Production in EMS Mutagenized Strains

**Figure 1:**
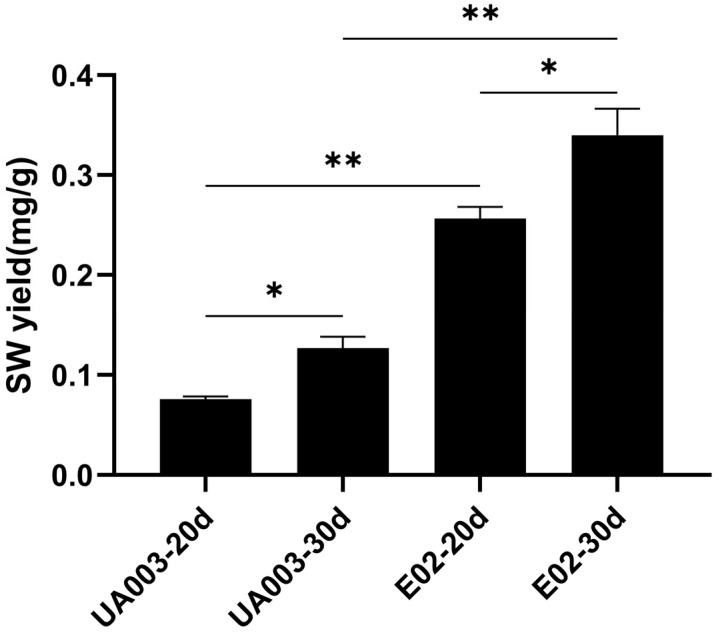
Swainsonine yield in the mycelium of wild and induced strains. The data represents the means ± SD of biological experiments conducted in triplicate. Statistical significance with * *p* < 0.05 and ** *p* < 0.01 was determined using non-parametric and parametric one-way ANOVA, respectively.

This study has indicated that the rate of protoplast regeneration decreased with higher concentrations of EMS. Mutated protoplasts did not survive when the EMS concentration was at 0.06 M or 0.08 M. However, colonies were able to survive on the regeneration medium at the 0.02 M and 0.04 M EMS concentrations. In this study, 34 colonies that were mutagenized by EMS and were able to grow in the regeneration medium were randomly selected. The mycelial SW content of each regenerated strain after the mutagenic treatment was compared with that of the wild-type strain without the mutagenic treatment. The study has found that the SW content in the 12 EMS mutagenized-treated strains that were regenerated was noticeably higher than in the wild-type strains that were treated with EMS at a working concentration of 0.02 M for 30 min (Supplementary Figure S1A). Furthermore, 12 strains obtained from initial screening in this study underwent three consecutive generations of passaging culture and SW assay. The mutagenized strains E02, E23, and E25 consistently exhibited significantly higher SW content than the wild-type strains after three consecutive generations of culture (Supplementary Figure S1B). We compared the levels of swainsonine in the mycelium of the wild-type strain *A. oxytropis* UA003 and the mutant strain E02 at 20 and 30 days of incubation. The results indicate a significant increase in swainsonine content in both the wild-type strain UA003 and the mutant strain E02 as the culture time was extended (*p* < 0.05), and the swainsonine content in the wild-type strain was significantly lower (*p* < 0.01) compared to the mutagenized E02 strain. Both isolates produced more SW after 30 days than after 20 days of culture (*p* < 0.05). And the isolate E02 produced more SW than UA003 under both culture time (*p* < 0.01) (Figure 1).

### 3.2. Analysis of Transcriptome Sequencing Quality Control Data

After conducting transcriptome sequencing using the HiSeq TM platform, the four sample groups produced three parallel samples with clean reads ranging from 40.18–59.48 million. The distribution of reads in each subgroup was as follows: 50.31–57.83 million in the C20 group, 40.18–57.81 million in the T20 group, 57.31–59.48 million in the C30 group, and 50.89–55.44 million in the T30 group. The T30 group produced 59.48 million reads, while the range for the T30 group was between 50.89 and 55.44 million. It is evident that the differentially expressed genes in the wild and mutant strains at various culture times are not identical, pointing to the multiplicity and complexity of the mechanisms of SW synthesis. The base quality values Q20 and Q30 in all samples’ clean data were over 95% and 88%, respectively, indicating they met the criteria for further analysis (Supplementary Table S2).

### 3.3. Differential Expression Gene Analysis

**Figure 2:**
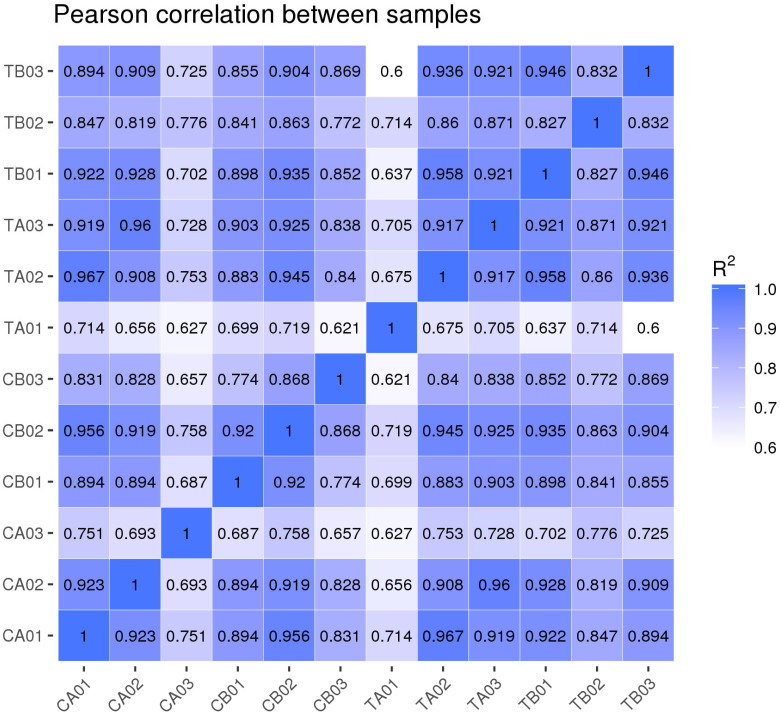
Correlation map of gene expression.

The average percentage of RNA-Seq reads that mapped to the reference genome exceeded 98% in all cases. Gene expression pattern correlation analysis (Figure 2) demonstrated noteworthy differences among samples and strong reproducibility between groups (Pearson correlation coefficients R^2^ > 0.9).

**Figure 3:**
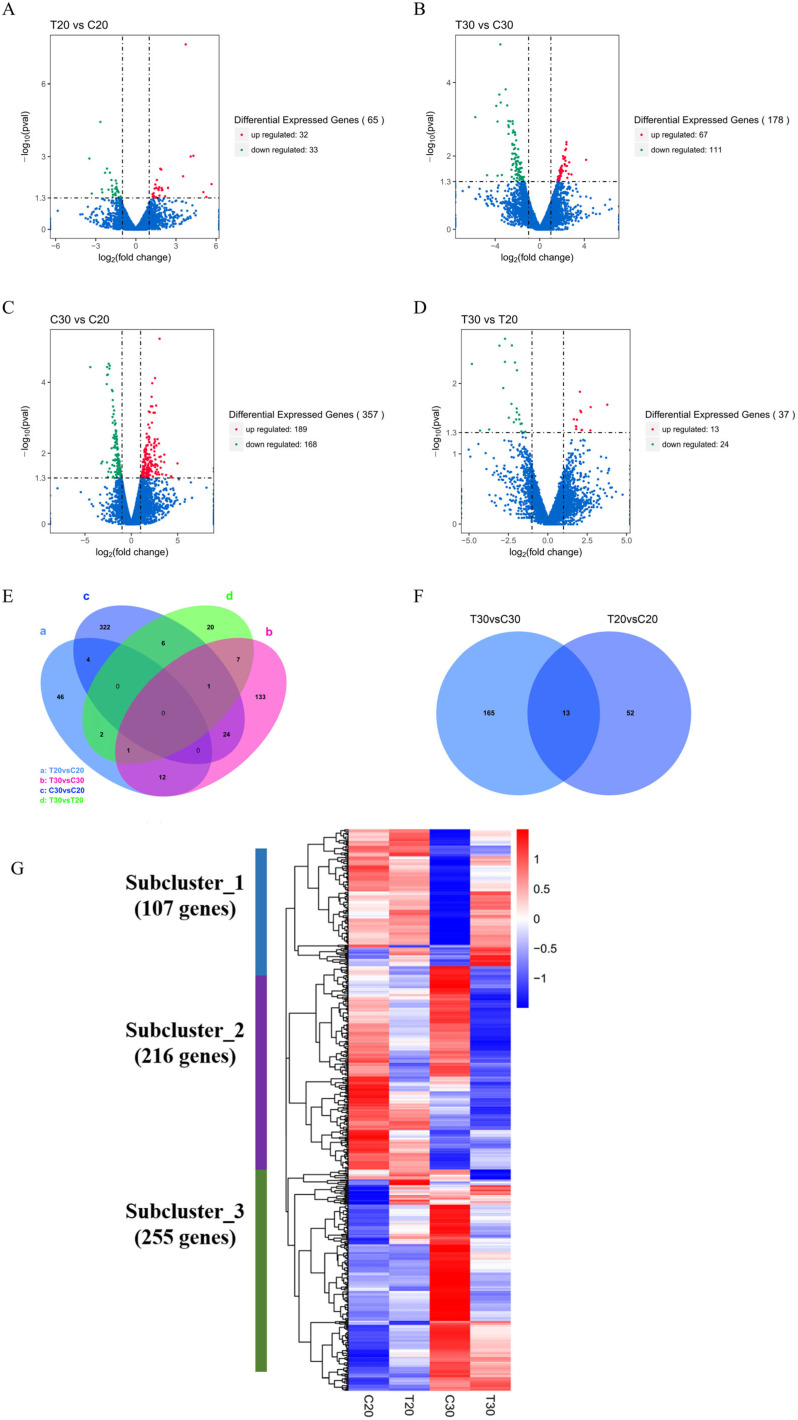
Volcano plots for differential mRNA expression analysis. (**A**) Differential analysis of mRNA expression between the C20 and T20 groups. (**B**) Differential analysis of mRNA expression between the C30 and T30 groups. (**C**) Differential analysis of mRNA expression between the C30 and C20 groups. (**D**) Differential analysis of mRNA expression between the T20 and C30 groups. (**E**) Differential analysis of mRNA expression differences between the T20 and C30 groups. Red indicates up-regulated differential genes, green indicates down-regulated differential genes, and blue indicates genes with non-significant differences. The horizontal coordinates show the fold change in gene expression in different samples, while the vertical coordinates indicate the statistical significance of the differences in gene expression changes. (**F**) Wayne presents plots showing the number of differential genes and overlapping genes resulting from comparing four different comparison groups. Additionally, Wayne provides plots of the number of differential genes and overlapping genes obtained by comparing the wild and mutagenized strains with varying incubation times. (**G**) Hierarchical clustering and cluster analysis of overlapping genes in a heat map.

First, we conducted a screening for differentially expressed genes by applying the combined criteria of a minimum 2-fold change and an ANOVA test (*p* < 0.05, FDR < 0.01). Next, we used FPKM values to calculate gene expression levels based on unique reads obtained from the four sample groups. Lastly, we compared changes in gene expression separately for the four sample groups. Data on gene expression were statistically analyzed to screen for genes displaying significant changes in expression across the various state samples. The significance of each gene was also evaluated in all comparative combinations. Based on the gene expression of wild and mutant strains at various culture times, a total of 637 differentially expressed genes, including overlapping and unique genes, were observed (Figure 3A–F). A total of 65 differentially expressed genes were observed in the T20 group compared to the C20 group, with 32 genes up-regulated and 33 genes down-regulated, including two genes enriched in the SW synthesis pathway. In the T30 group, there were 178 genes that demonstrated differential expression in comparison to the C30 group. This included 67 up-regulated genes and 111 down-regulated genes, as well as 14 genes that were enriched for the SW synthesis pathway. A total of 357 genes, with 189 up-regulated and 168 down-regulated, were differentially expressed in the C30 group compared to the C20 group. Among them, 7 genes were enriched for the SW synthesis pathway. A total of 37 genes that exhibited differential expression were identified in the T30 group as compared to the T20 group. Out of these, 13 genes were noted to be up-regulated, while 24 genes were down-regulated. Additionally, 5 genes that were enriched for the SW synthesis pathway were identified. Under different culturing time conditions, we noted for the four sample groups that the number of differential genes that overlapped was 13 for the mutant strain in comparison to the wild strain at 20 and 30 days of culture.

Differential gene clustering analysis based on FPKM values yielded a total of 578 differential genes, and through the clustering algorithm, we analyzed the trends of expression levels of differential genes and plotted the heat maps of the levels of these transcripts (Figure 3G). These genes with differential expression can be classified into three gene clusters, as follows: Genes in gene cluster 1 show a trend of down-regulation as the SW content increases in the wild strain and significant up-regulation as the SW content increases in the mutant strain. Genes in gene cluster 2 show a significant down-regulation with increasing SW content in both the wild and mutant strains. Genes in gene cluster 3 indicate an up-regulation trend with increasing SW content in the wild strain but significant down-regulation as the SW content increases in the mutant strain. These findings imply that genes associated with SW content are more commonly present in the cluster 2 group.

### 3.4. Alternative Splicing Events Detected in the A. oxytropis Transcriptome

Alternative splicing plays a crucial regulatory role in the growth of fungi. To investigate the impact of selective splicing on gene expression in the endophytic fungus *A. oxytropis* in locoweed, we examined wild-type and mutant strains for alternative splicing. We identified two forms of alternative splicing, Skipped exon (SE) and Mutually exclusive exons (MXEs), and did not come across instances of alternative 5′ splice site selection (A5SS), alternative 3′ splice site selection (A3SS), or intron retention (RI). We analyzed the differences for each category of variable shear events separately and found that AS events exhibited significant changes among groups, considering FDR < 0.05 as the screening criterion for differential AS events. Specifically, alternative splicing events involving skip exons (SEs) were found in 372 genes and mutually exclusive exons (MXEs) in 21 genes in the T20 group, compared to the C20 group. In the T30 group, SEs appeared in 514 genes and MXEs in 30 genes, compared to the C30 group, where SEs appeared in 507 genes and MXEs in 33 genes. Furthermore, compared to the T20 group, the T30 group exhibited SEs in 387 genes and MXEs in 14 genes. We observed that alternative splicing (AS) commonly occurs in four genes, indicating a conserved splicing mechanism in *A. oxytropis*. These genes were evm.TU.Contig1.330, evm.TU.Contig30.151, evm.TU.Contig46.13, and evm.TU.Contig65.51. Due to the high number of AS events, these findings suggest a complex splicing pattern in both wild-type and mutant *A. oxytropis* strains during SW production changes.

### 3.5. GO Enrichment Analysis

**Figure 4:**
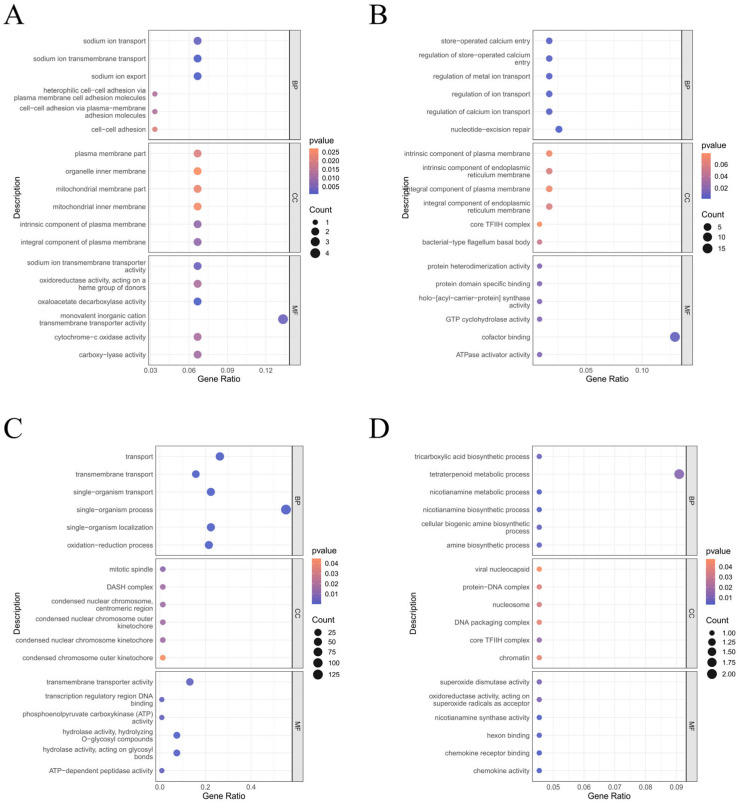
GO categorization of the unigene distribution of genes in different GO function classifications. (**A**) T20 group vs. C20 group, enriched for differentially expressed genes in the GO pathway. (**B**) T30 group vs. C30 group, enriched for differentially expressed genes in the GO pathway. (**C**) C30 group vs. C20 group, enriched for differentially expressed genes in the GO pathway. (**D**) T30 group vs. T20 group, enriched for differentially expressed genes in the GO pathway. For the annotations in the figure, BP is the biological process, CC is the cellular component, and MF is the molecular function. Red indicates up-regulation of gene expression, while blue indicates down-regulation of gene expression.

To investigate the biological functions of the identified genes, we obtained a total of 7100 differential genes from the screening. We then comparatively analyzed 1212 proteins with E-values ≤ 1 × 10^−5^ to determine the detailed functions of the differential proteins found in *A. oxytropis*. To annotate the functions of these proteins, we utilized GO terms from TAIR (http://www.arabidopsis.org/tools/bulk/go/index.jsp, accessed on 23 January 2023). The statistically summarized proportion of differentially up-regulated and down-regulated genes was determined among the three principal GO classifications: molecular function (MF), biological process (BP), and cellular component (CC). GO enrichment has shown that the screened 7100 DEGs were enriched into 1212 GO pathways, and the 30 GO terms with the most significant enrichment were subsequently selected and plotted in bar graphs as shown in Figure 4. Pathways involved in molecular functions in the T20 group compared to the C20 group, including oxidoreductase activity (GO:0016491) enriched into 9 differentially expressed genes, monovalent inorganic cation transmembrane transporter protein activity (GO:0015077) enriched into 4 differentially expressed genes, monovalent inorganic cation monomer (GO:0015077) enriched into 4 differentially expressed genes, hydrogen ion transmembrane transporter (GO:0015078) enriched into 3 differentially expressed genes, oxaloacetate dehydrogenase (GO:0008948), sodium ion transmembrane transporter (GO:0035725), and carboxyl cleavage enzyme activity (GO:0016831) pathways were significantly enriched (Figure 4A). Pathways involved in molecular function in the T30 group compared to the C30 group, including cofactor binding (GO:0048037) enriched into 15 differentially expressed genes, ncRNA treatment (GO:0034470) enriched into 7 differentially expressed genes, nucleotide-excision repair (GO:0006289), regulation of metal-ion conversion (GO:0010959), ion transport regulation (GO:0043269), and bile acid and bile salt transport (GO:0015721) pathways were significantly different (Figure 4B). Pathways involved in biological processes, including single organism transport (GO:0044765), were enriched into 62 differentially expressed genes in the C30 group as compared to the C20 group, and redox processes (GO:0055114) were enriched into 52 differentially expressed genes. Pathways such as the single organism metabolic process (GO:0044699) were significantly enriched, and pathways involving molecular functions, including carbohydrate metabolism transmembrane transporter activity (GO:0005975) and transporter activity (GO:0005215), were significantly different (Figure 4C). The pathways involving molecules, including oxidoreductase activity (GO:0055114) (acting as a receptor for superoxide radicals), were enriched into 7 differentially expressed genes, and pathways involving biological processes, including tetraterpene metabolism (GO:0016108) enriched into 3 differentially expressed genes and tetraterpene biosynthesizers (GO:0016109), as compared to group T30 vs. T20, flavin adenine dinucleotide synthesis (GO:0050660), core TFIIH complex (GO:0000439), and amine biosynthesis (GO:0030418) pathways, were significantly different (Figure 4D). These metabolic pathways involve metabolic processes related to the growth, reproduction, and accumulation of secondary metabolites in *A. oxytropis*, suggesting that these metabolic pathways are closely related to the entire developmental process of the *A. oxytropis* fungus. These results suggest that *A. oxytropis* undergoes a dynamic change in the synthesis of SW that produces differences in SW content.

### 3.6. KEGG Pathway Analysis

**Figure 5:**
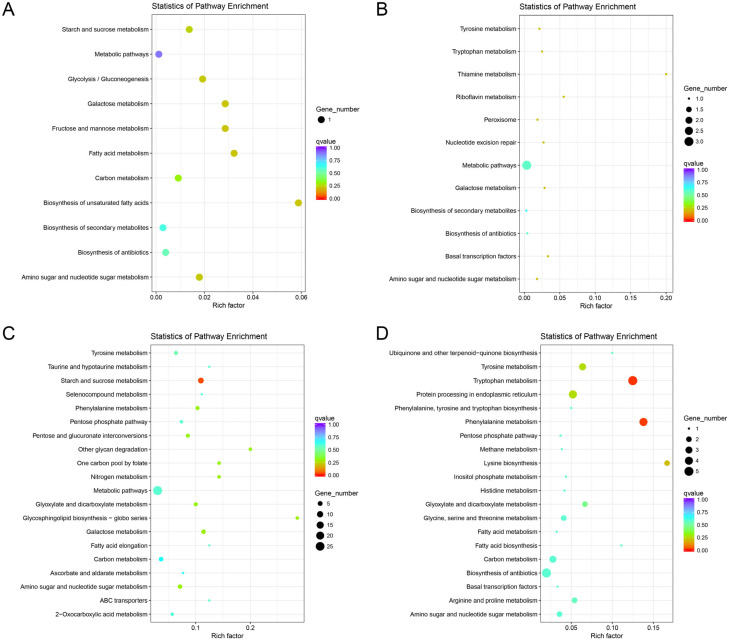
KEGG annotation of putative proteins. Distribution of genes in different KEGG categories. (**A**): T20 group vs. C20 group, enriched for differentially expressed genes in the KEGG pathway. (**B**): T30 group vs. C30 group, enriched for differentially expressed genes in the KEGG pathway. (**C**): C30 group vs. C20 group, enriched for differentially expressed genes in the KEGG pathway. (**D**): T30 group vs. T20 group, enriched for differentially expressed genes in the KEGG pathway. Red indicates up-regulation of gene expression, while blue indicates down-regulation of gene expression.

To determine whether differentially expressed genes are involved in specific pathways, we annotated 637 differentially expressed genes using KEGG blast and homology enriched them into the AGI locus of *A. oxytropis*. A scatterplot of the results of the KEGG enrichment analysis is shown in Figure 5. In this figure, the degree of KEGG enrichment is measured by the Rich factor, the Q value, and the number of genes enriched by this pathway. We selected the 20 pathway entries with the most significant enrichment to be shown in this figure. Among them, the biosynthesis of unsaturated fatty acids (bor01040), fatty acid metabolism (bor01212), galactose metabolism (bor00052), and fructose and mannose metabolism (bor00051) pathways contained the most differentially expressed genes.

Specifically, one key differential gene was associated with significant enrichment of biosynthesis of unsaturated fatty acids (bor01040), fatty acid metabolism (bor01212), fructose and mannose (bor00051), glycolysis/gluconeogenesis (bor00010), biosynthesis of secondary metabolites (bor01110), and metabolism (bor00051) in the T20 group compared to the C20 group, respectively (Figure 5A). In the T30 group compared to the C30 group, one differential gene was associated with significantly enriched 2-oxocarboxylic acid metabolism and fatty acid biosynthesis (bor00061) and inositol phosphate metabolism (bor00562) pathways, respectively. In the biosynthesis of amino acids (bor01230) and lysine biosynthesis (bor00300) pathways, respectively, two differential genes were associated with significant enrichment in the tryptophan metabolism (bor00350) pathway, and four differential genes were associated with the significantly enriched phenylalanine metabolism (bor00360) pathway (Figure 5B). The pathways enriched in the KEGG analysis in the wild and mutant strains under the same culture time conditions were mostly involved in the P6C pathway of SW synthesis. Two differential genes were associated with significant enrichment in the glycosphingolipid biosynthesis (bor00603) pathway and three differential genes with significant enrichment in the tyrosine metabolism (bor00350) and phenylalanine metabolism (bor00360) pathways in the C30 group compared to the C20 group, respectively (Figure 5C). Four differential genes were associated with the amino sugar and nucleotide sugar metabolism (bor00520) and galactose metabolism (bor00052) pathways. Likewise, 30 differential genes were associated with the significantly enriched metabolic pathway (bor01100). One differential gene was associated with significant enrichment of biosynthesis of secondary metabolites (bor01110) and amino sugar and nucleotide sugar metabolism (bor00520) in the T30 group compared to the T20 group, respectively (Figure 5D). The pathways of KEGG enrichment in wild and mutant strains under different culture time conditions were mostly associated with the strain’s own metabolism.

### 3.7. Synthesis Pathway of SW in A. oxytropis

**Figure 6:**
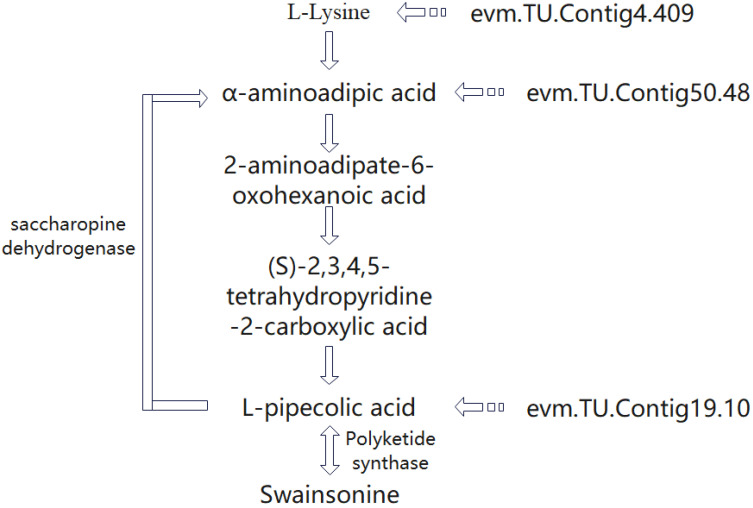
Key gene sites affecting SW synthesis in the locoweed endophytic fungus *A. oxytropis*.

The indolizidine alkaloid SW, as a compound with a relatively simple chemical formula, is obtained within *A. oxytropis* mainly by the conversion of L-piperidinic acid, and the pathway for the production of L-piperidinic acid is currently thought to be synthesized by the P6C pathway within the fungus [23,24]. The evm.TU.Contig4.409, evm.TU.Contig19.10, and evm.TU.Contig21.92 genes are enriched in the pathways of L-lysine synthesis and metabolism, piperidinic acid synthesis and degradation, and α-aminoadipic acid. The P6C pathway is the main pathway for SW synthesis, specifically indicated as: L-Lysine → α-aminoadipic acid → 2-aminoadipate-6-oxohexanoic acid → (S)-2,3,4,5-tetrahydropyridine-2-carboxylic acid → L-pipecolic acid. L-pipecolic acid, as a key product in the P6C pathway, is a catabolic product of lysine, which is catalyzed by saccharopine dehydrogenase to generate α-aminoadipic acid in the P6C pathway and also catalyzed by polyketide synthase to form SW by P450 enzyme catalysis (Figure 6).

### 3.8. RT-qPCR Analysis of Genes Associated with SW Synthesis in Mycelium

**Figure 7:**
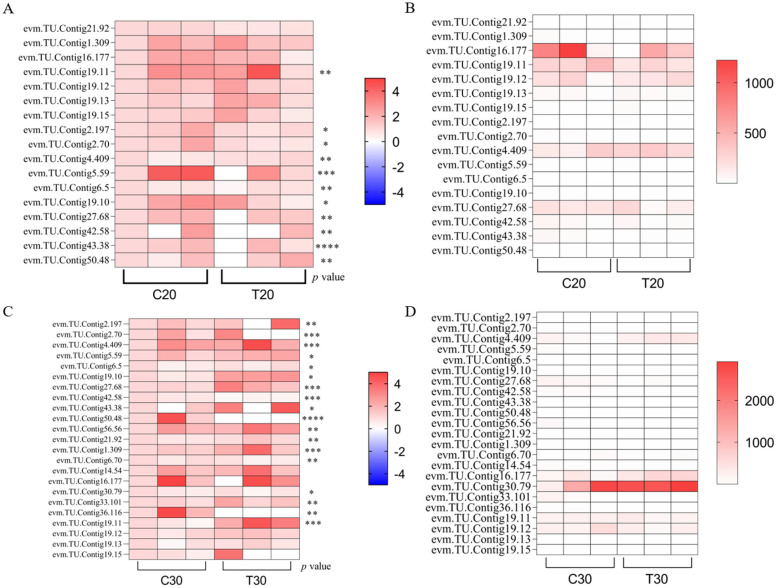
Expression profiles of 17 SW biosynthesis-related unigenes in *A. oxytropis*. (**A**) Changes in relative expression levels of mRNA genes associated with SW synthesis in the T20 group compared to the C20 group (N = 3). (**B**) Transcriptome analysis of the T20 group compared to the C20 group, FPKM values of mRNA genes associated with SW synthesis (N = 3). (**C**) Changes in relative expression levels of mRNA genes associated with SW synthesis in the T30 group compared to the C30 group (N = 3). (**D**) Transcriptome analysis of the T30 group compared to the C30 group, FPKM values of mRNA genes associated with SW synthesis (N = 3). All data from biological experiments performed in triplicate are presented as means ± SD. * *p* < 0.05, ** *p* < 0.01, *** *p* < 0.001, and **** *p* < 0.0001, determined using non-parametric one-way ANOVA.

To quantitatively determine the reliability of our transcriptome data, we examined the expression of 23 candidate differential genes using RT-qPCR assessment methods and analyzed them statistically by comparing them with the FPKM results. The results showed that a total of 11 key genes that may be involved in SW synthesis were screened. In the T20 group compared to the C20 group, significant up-regulation of genes such as evm.TU.Contig4.409, evm.TU.Contig19.10, evm.TU.Contig43.38, and evm.TU.Contig42.58 took place, and significant down-regulation of genes such as evm.TU.Contig19.11, evm.TU.Contig50.48, and other genes underwent significant down-regulation. In the T30 group compared to the C30 group, evm.TU.Contig4.409, evm.TU.Contig21.92, evm.TU.Contig1.309, evm.TU.Contig16.177, and evm.TU.Contig19.10 genes underwent significant up-regulation, while evm.TU.Contig6.70 and evm.TU.Contig50.48 underwent significant down-regulation. By analyzing the SW content assay, we found that, compared with the blank control group, in the T20 and T30 groups, the expression of evm.TU.Contig4.409 and evm.TU.Contig19.10 genes showed a continuous up-regulation with the increase in SW content, while the expression of the evm.TU.Contig50.48 gene showed a continuous down-regulation, suggesting that these three genes might be associated with the key steps involved in SW synthesis (Figure 7).

## 4. Discussion

Swainsonine is a toxic secondary metabolite produced by locoweed. It inhibits α-mannosidase activity and poisons animals [25]. However, it also has anti-tumor and immunomodulatory effects. The biosynthetic pathway of SW in fungi still remains unclear. For the first time, we sequenced the wild strain and EMS-induced mutant strains of endophyte *A. oxytropis* under different culturing time conditions using the Illumina HiSeqTM4000 high-throughput sequencing platform, with the aim of exploring the biosynthetic pathway of SW.

He et al. [19] analyzed the transcriptome of *Oxytropis ochrocephala* (containing locoweed toxin) under drought, salt, and cold stress and identified several genes that might be involved in drought, salt, and cold domestication, but did not functionally analyze these candidate genes. With further research into possible candidate genes for SW yield, Cook et al. investigated the biosynthetic genes, i.e., *SwnA*, a putative aminotransferase, and *SwnT*, for swainsonine in a variety of commensal and pathogenic fungi, which is associated with transmembrane choline transporter proteins, and edited the target genes to validate the gene functions. Researchers have found that the wild strains exhibit slightly lower virulence than the knockout strains, suggesting that expression of the *SwnA* and *SwnT* genes indeed affects the yield of SW [9,26,27]. With the rapid development of bioinformatics technology, it is necessary for researchers to realize a reliable and in-depth screening of genes determining SW production in endophytic fungi in locoweed by applying more advanced technological means [28].

Swainsonine is influenced by a variety of factors, and in this experiment, we used EMS for continuous in vitro induction of the endophytic fungus *A. oxytropis*. When choosing different EMS concentrations, we found that the growth of the wild-type strain was inhibited as the EMS concentration increased, and the wild-type strain could not grow normally when EMS was used at concentrations of 0.06 mol/L and 0.08 mol/L. When using working concentrations of EMS of 0.04 mol/L and 0.02 mol/L, we found that the working concentration of 0.02 mol/L was the most effective in increasing the amount of SW as the incubation time increased. Analyzing the reasons, we believe that the addition of EMS affects the growth and metabolism of the wild-type strain itself, causing irreversible damage to the strain [29]. By measuring the SW concentration of strain E02, we found that the SW content shows a positive correlation with the number of days of culture, which increased as the number of days of culture increased [30]. Meanwhile, we found that in the blank control group UA003 strain, the content of SW still showed an increasing trend with the increase in the number of days of culture, which indicates that the wild-type strains also accumulated SW content with the increase in the number of days of culture under the condition of no external stimulation.

Wu C et al. [15] analyzed SW phytotoxicity and explored the mechanism of poisoning after consumption by the livestock. Noor et al. [27] further explored the synthetic pathway of SW through the analysis of the fungal ketide synthase gene. Guan et al. [31] investigated the potential antitoxicity of the endophytic fungi in locoweed, which was found to possess certain anthelmintic effects. With the gradual deepening of the understanding of SW, researchers have not only analyzed the function of SW but also proposed further thoughts on the pathway of SW synthesis [32]. The analysis of the SW synthesis relationship of endophytic fungi in locoweed showed that the number and synthesis ability of endophytic fungi in plant tissue genes in locoweed had a direct relationship with the content of SW [33]. A large number of endophytic fungi, such as *Eriges* spp., that can be produced synthetically are present in locoweed. Due to the growth characteristics of locoweed plants, the number of endophytic fungi varies in different growth areas and locations [34].

Endophytic fungal transcriptome sequences are abundant, and database systems were applied to identify gene sequences and analyze their synthetic genes, a large number of which were not annotated and could not be matched with corresponding information in the database [10]. The information on the gene sequences of endophytic fungi in locoweed studied by scholars from various countries has not yet been fully published, and the synthesis pathway is affected by the gene sequences, which does not allow for a complete annotation of the SW gene [35]. It is known that endophytic fungi in locoweed can produce saccharopine reductase under the catalytic action of the saccharopine reductase gene and can synthesize SW under the action of gene metabolism [36]. In the fungal SW biosynthesis pathway, L-lysine generates α-aminoadipic acid, which ultimately forms L-pipecolic acid, one of the precursors of SW biosynthesis. Therefore, the levels of L-lysine and α-aminoadipic acid are major factors in the amount of SW biosynthesis. Significant up-regulation of evm.TU.Contig4.409 occurred in the T20 group compared to the C20 group and in the T30 group compared to the C30 group. The expression of evm.TU.Contig19.10 was significantly higher in the T30 group than in the C30 group, whereas evm.TU.Contig50.48 expression was significantly lower in the T30 group than in the C30 group. The screening of these genes excluded the effect of the incubation time of the *A. oxytropis* strain on SW content. Therefore, we hypothesized that altered gene expression of evm.TU.Contig4.409, evm.TU.Contig50.48, and evm.TU.Contig19.10 resulted in the accumulation of L-lysine and α-aminoadipic acid content, which caused an increase in the amount of SW biosynthesis. L-lysine is an essential amino acid within fungi and plays an important role in fungal biosynthetic processes and SW biosynthesis. Meanwhile, by analyzing the relationship between key differential genes and SW content, we screened and obtained three key genes. The expression of these genes showed positive or negative regulation with the increase in SW content, and it was hypothesized that these genes were involved in the process of SW synthesis. In this study, 23 of the DEGs involved in the L-lysine biosynthesis and degradation pathway were annotated in the KEGG pathway, but some of the differential genes involved still had unannotated functions, and their expressions were significantly changed. All of this provides evidence for the search for key genes for SW synthesis.

In this study, we used the wild-type strain *A. oxytropis* UA003 and the induced strain E02 strains for transcriptomic analysis, and combined with the RT-qPCR results, 11 differential genes were obtained from the screening, of which evm.TU.Contig4.409, evm.TU.Contig19.10, and evm.TU.Contig50.48 were enriched to L-lysine synthesis and metabolism, L-pipecolic acid synthesis and degradation, and α-aminoadipic acid, which can be used as candidate genes to clarify the synthesis pathway of SW of endophytic fungi in locoweed. Meanwhile, we found that eight genes, including evm.TU.Contig1.309, evm.TU.Contig42.58, evm.TU.Contig19.11, and evm.TU.Contig16.117, were not functionally annotated in the *A. oxytropis* strain and were not enriched in any possible metabolic pathways, which provides new data support for finding the SW synthesis pathway and needs to be further investigated. The development of this study can promote the key link from theoretical research to application to reduce or eliminate the toxicity of locoweed from the perspective of controlling the endophytic fungi of locoweed and efficiently producing SW from the perspective of biosynthesis.

## Data Availability

The transcriptome data of the fungi under study have been uploaded to the NCBI GenBank Sequence Read Arxchive (SRA) database under accession number PRJNA1017857. The data set generated and analyzed during this study can be obtained from the corresponding authors upon reasonable requirements.

## Supplementary Materials

The following supporting information can be downloaded at: https://www.mdpi.com/article/10.3390/jof10010088/s1, Figure S1: Swainsonine yield in mycelium of wild and induced strains.; Table S1: List of primers used in RT-qPCR; Table S2: Quality of data outputs.
