# Supplementary material for: Transcriptomic Screening of Alternaria oxytropis Isolated from Locoweed Plants for Genes Involved in Mycotoxin Swainsonine Production
Source: J Fungi (Basel). 2024 Jan 22;10(1):88. doi: 10.3390/jof10010088 (PMC10820250; doi:10.3390/jof10010088)
Supplement: Supplementary file 1 [file jof-10-00088-s001.zip › jof-2793599-supplementary.pdf]

Supplementary Materials:

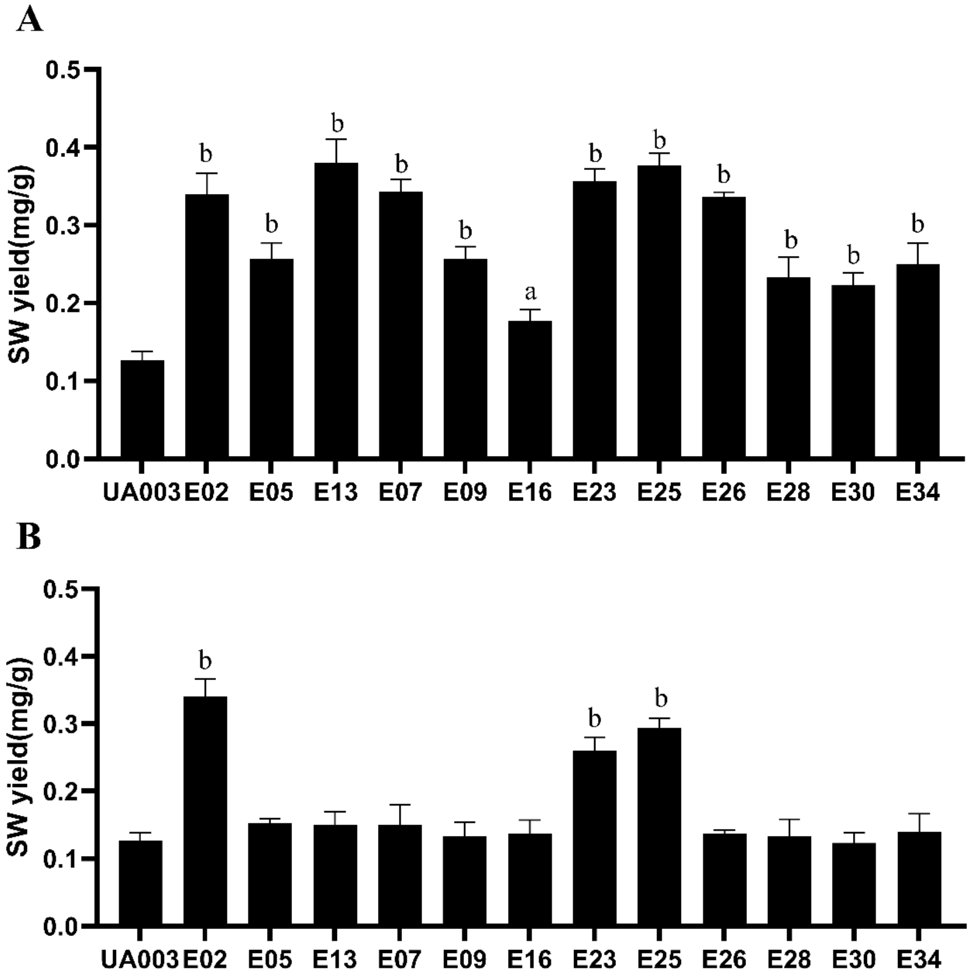

Supplementary Figure S1 Swainsonine yield in mycelium of wild and induced strains. A: The SW yield of wild type UA003 strain and each mutant strain after 30 minutes of EMS mutagenesis. **B**: The SW yield of wild type UA003 strain and each mutagenic strain after EMS mutagenesis after 30 days of culture. The data represents means ± SD from biological experiments conducted in triplicate. Statistical significance with [a] $p < 0.05$ and [b] $p < 0.01$ was determined using non-parametric and parametric one-way ANOVA, respectively.

Supplementary Table S1. List of primers used in RT-qPCR.

Gene name	Primers	Sequence (5′- 3′)	Product Size (bp)
GAPDH-F	Forward primer	GCCGGTATCTCTCTCAACAAG	100 bp
	Reverse primer	CAATGTAGACCAGGAGGTCAAG	
evm.TU.Contig 2.197	Forward primer	TTCTCAGGAAGCGGTGGTG	106 bp
	Reverse primer	CGGGTGGGGTATTGTCATTT	
evm.TU.Contig 2.70	Forward primer	TTGTCTACGATGACGGAAACC	90 bp
	Reverse primer	AGAAACACTCTGACGCTGTAATTG	
evm.TU.Contig 4.409	Forward primer	TTGGTTATTACTATTCTCGGTGGAC	249 bp
	Reverse primer	GGTGCTCATTGCTGGGTTATC	
evm.TU.Contig 5.59	Forward primer	CGTGCCAACATCGGAAAC	175 bp
	Reverse primer	GGGAAATGATAGGAATGAACCA	
evm.TU.Contig 6.5	Forward primer	GCCTAAGCGATGCGAAGA	98 bp
	Reverse primer	CAGTGGGGAGGTTGGAGAG	
evm.TU.Contig 19.10	Forward primer	CAGTGATGCACGGAGGATATAG	107 bp
	Reverse primer	GCTATTCGGTTCCAAGACAGA	
evm.TU.Contig 27.68	Forward primer	TGAAGGCGAAGATTTAGAAGTTATT	128 bp
	Reverse primer	CTATGATGTAGAGGGCGAGATTG	
evm.TU.Contig 42.58	Forward primer	AAACTGCTCGTGGATCGCCTTTC	125 bp
	Reverse primer	TCGTAAGAATCGGTTCGTGTGCTG	
evm.TU.Contig 43.38	Forward primer	CAGGTTGGGGCGTCTACTTT	87 bp
	Reverse primer	CAGTGTAGGAACGGCTGCTTT	
evm.TU.Contig 50.48	Forward primer	TAGGCTCGTCGCTTGTGG	101 bp
	Reverse primer	ACAGCAAGGCACCTGGAAT	
evm.TU.Contig 56.56	Forward primer	GGTTACCTCGACTGGCTAATTC	99bp
	Reverse primer	CATGACTTCTGTGGGCAGAT	
evm.TU.Contig 21.92	Forward primer	TTACTGCTGGACAACCATCC	109 bp
	Reverse primer	GCATCACACCTTCCTTCTCTAA	
evm.TU.Contig 1.309	Forward primer	CTCCAACAGGCTATGGCTAACTA	113 bp
	Reverse primer	AATCCTCGGTGGGGAAGTG	
evm.TU.Contig 6.70	Forward primer	ACATTGTTGAAAGACACCGACC	90 bp
	Reverse primer	CTGCTGACGCTCACTTGGAT	
evm.TU.Contig 14.54	Forward primer	CCAGGTATGCGGCAAAGAA	248 bp
	Reverse primer	TCGGTTGGGTAGGGGTGA	
evm.TU.Contig 16.177	Forward primer	TCTCTCATCTCCAACCAACTTCA	143 bp
	Reverse primer	TTCCAAATGTTATCACCCACG	
evm.TU.Contig 30.79	Forward primer	CTTCGCCGAGATTGACCC	218 bp
	Reverse primer	TTGGGTTCATCTGGTTCACG	
evm.TU.Contig 33.101	Forward primer	CTACTACAAGCAAGACCCCAAAC	154 bp
	Reverse primer	GCCACTTCCACCTCCAACA	
evm.TU.Contig 36.116	Forward primer	ACGCAAATGGGTCAGGAAGT	108 bp
	Reverse primer	ACGAGTACGCAGGATGGGA	
evm.TU.Contig 19.11	Forward primer	GACAACGCCTCCATCTCTTT	119 bp
	Reverse primer	CTCGCTTGCTACCTGCTATC	

evm.TU.Contig 19.12	Forward primer	GGAACCAGGTGATGCATTTG	105 bp
	Reverse primer	GAGATGAAGAAGAGGCCGTATAG	
evm.TU.Contig 19.13	Forward primer	GTTGTCGTTGCTGTCGCC	99 bp
	Reverse primer	ATGTTTCGTCTTGTTGGTTGC	
evm.TU.Contig 19.15	Forward primer	GTCCGGGTATGTCTTGATGTT	120 bp
	Reverse primer	GACACATCGCCTGCCTATAC	

Supplementary Table S2. Quality of data outputs.

Sample name	Clean reads	Q20（%）	Q30（%）
CA01	57839364	96.17	90.48
CA02	51342636	95.91	89.71
CA03	50319638	96.73	91.52
TA01	40186148	96.13	90.40
TA02	50117874	96.29	90.45
TA03	57814064	95.44	88.80
CB01	59483678	95.68	89.28
CB02	57311310	96.78	91.67
CB03	59358510	95.82	89.51
TB01	53186892	96.77	91.60
TB02	55440578	96.72	91.44
TB03	50891492	97.07	92.22
